# Validity and reliability of healthy food knowledge and healthy food preferences scale for preschool children

**DOI:** 10.3389/fped.2025.1507055

**Published:** 2025-04-03

**Authors:** Aklilu Abrham Roba, Öznur Başdaş

**Affiliations:** ^1^ College of Health and Medical Sciences, Haramaya University, Dire Dawa, Ethiopia; ^2^ Nursing Department (Child Health and Diseases Nursing Program), Institute of Health Sciences, Erciyes University, Kayseri, Türkiye; ^3^ Faculty of Health Sciences, Erciyes University, Kayseri, Türkiye

**Keywords:** healthy food knowledge (HFK_PS), healthy food preference (HFP_PS), preschool children, reliability, validity

## Abstract

**Introduction:**

Early childhood nutrition plays a critical role in shaping lifelong health outcomes, yet preschool children in low- and middle-income countries often have poor dietary habits and limited knowledge of healthy foods. This study aimed to validate and assess the reliability of the 9-item Healthy Food Knowledge (HFK_PS) and Healthy Food Preference (HFP_PS) scales, ensuring cultural relevance for measuring food knowledge and preferences among Ethiopian preschool children aged 3–7 years.

**Methods:**

A cross-sectional design was employed, involving 319 preschoolers from five randomly selected 5 kindergarten schools. Data was collected through an interactive photo-based interview with the children. A panel of ten experts assessed content validity, while construct validity was assessed using Exploratory Factor Analysis (EFA) and Confirmatory Factor Analysis (CFA). Reliability was assessed using Cronbach's alpha.

**Results:**

The EFA identified a two-factor structure for both scales, explaining 50.91% and 50.18% of the variance for HFK_PS and HFP_PS, respectively. CFA confirmed the model fit, with all indices meeting the recommended thresholds. The HFK_PS and HFP_PS scales demonstrated good internal consistency, with Cronbach's alpha values of 0.80 and 0.78, respectively. Older children and those in higher grades had significantly greater food knowledge (*p* < 0.001), and food preferences also improved with grade level (*p* < 0.05). However, preferences remained stable across age groups (*p* = 0.928). However, no significant gender differences were found. A positive correlation (*r* = 0.43, *p* < 0.001) was found between healthy food knowledge and preferences, suggesting that increased knowledge is associated with healthier preferences.

**Conclusions:**

The validated scales can be instrumental in evaluating preschoolers' dietary knowledge and preferences in Ethiopia. Future studies should focus on implementing these scales in nutrition education programs to assess their effectiveness in fostering long-term healthy eating habits among young children.

## Key messages

• The study successfully validated the Healthy Food Knowledge (HFK_PS) and Healthy Food Preference (HFP_PS) scales for preschool children using culturally relevant food items in Ethiopia. The scales showed strong internal consistency and validity.
• A positive correlation was found between healthy food knowledge and preferences among preschool children, suggesting that improved knowledge can influence healthier food preferences.
• Healthy food knowledge increased significantly with age and grade level, while preferences showed no significant change with age but improved with higher grade levels.
• There were no significant gender differences in healthy food knowledge or preferences.

## Introduction

Dietary patterns among preschool children in Ethiopia are shaped by socioeconomic, cultural, and environmental factors. Many children in low- and middle-income countries (LMICs), including Ethiopia, have poor dietary diversity. They rely on staple foods and consume fewer macro- and micronutrient-rich foods like fruits, vegetables, and animal-source foods (1, 2). Early childhood nutrition plays a key role in long-term health, cognitive development, and disease prevention (3–5).

Studies revealed that when parents and children possess low healthy food knowledge, there is a heavy reliance on monotonous staple foods, leading to child malnutrition (1, 6, 7). Malnutrition affects one in three people worldwide (8). Although the causes of malnutrition are complex and multilayered, dietary choices matter for optimal growth, health, and development (9). Dietary adequacy in children requires they are not only given access to but also agree to consume a variety of foods from different food groups to satisfy the energy, macro-, and micronutrient demands (10).

Preschool children are at the highest risk of malnutrition related to increased demand for growth and development, poor dietary diversity, repeated illness, etc. (11). On the other hand, children who are already overweight at preschool age bear a high risk of being obese at school age (12). Thus, feeding practices in early childhood are building blocks for healthy eating behavior later in life (3–5). Preschool age is the best time to intervene because behaviors that lead to a positive energy/nutrient balance are established during this period (13).

Developing culturally appropriate tools to assess and promote healthy food knowledge and preferences among preschool children is vital. This study focuses on the adaptation and validation of the Healthy Food Knowledge (HFK_PS) and Healthy Food Preference (HFP_PS) scales for preschool children in Harar, Ethiopia. These tools are adapted from the iPad activity initially developed to evaluate children's food and physical activity knowledge in Western contexts (14).

Age, grade level, and gender may influence food knowledge and preferences, but evidence remains inconclusive, warranting further research. While some studies suggest that nutrition knowledge and preferences improve with age and educational exposure (15, 16), the extent and consistency of these improvements remain uncertain. Similarly, research indicates that higher grade levels may enhance dietary knowledge due to increased health education exposure (17, 18), yet the variability across different educational contexts needs further exploration. Gender differences in healthy food knowledge and preferences are particularly mixed; while some studies found that girls might have better nutrition knowledge and healthier preferences, potentially due to sociocultural factors (15, 19), other studies report minimal gender differences, suggesting context-specific influences (20).

Cultural traditions, socioeconomic status, and food availability have a big impact on the eating habits of preschoolers in Ethiopia. This necessitates the modification of dietary assessment tools to ensure their accuracy and usefulness in this context. Traditional Ethiopian foods like injera, kinche, and atmit are important for health and culture, but they aren't often included in global dietary assessment tools. Instead, they often include processed cereals, packaged snacks, and Western foods that many Ethiopian children don't know or can't get. Additionally, socioeconomic disparities and seasonal food availability play a crucial role in shaping dietary choices, with many families relying on monotonous diets dominated by staple grains, contributing to high rates of child malnutrition and micronutrient deficiencies (1, 9).

Without culturally appropriate tools, tests may provide inaccurate information about children's nutrition knowledge and preferences. Such results could lead to ineffective policy recommendations and missed chances for early childhood nutrition interventions (19). Additionally, differences in age, grade level, and gender may influence healthy food knowledge and preferences, but current evidence remains inconclusive (21). This uncertainty underscores the necessity for targeted research to better understand how these factors impact dietary knowledge and choices. Beyond validating the HFK_PS and HFP_PS scales, this study aims to address these gaps by examining the role of age, grade level, and gender in shaping healthy food knowledge and preferences among Ethiopian preschool children.

## Methods

**Figure 1:**
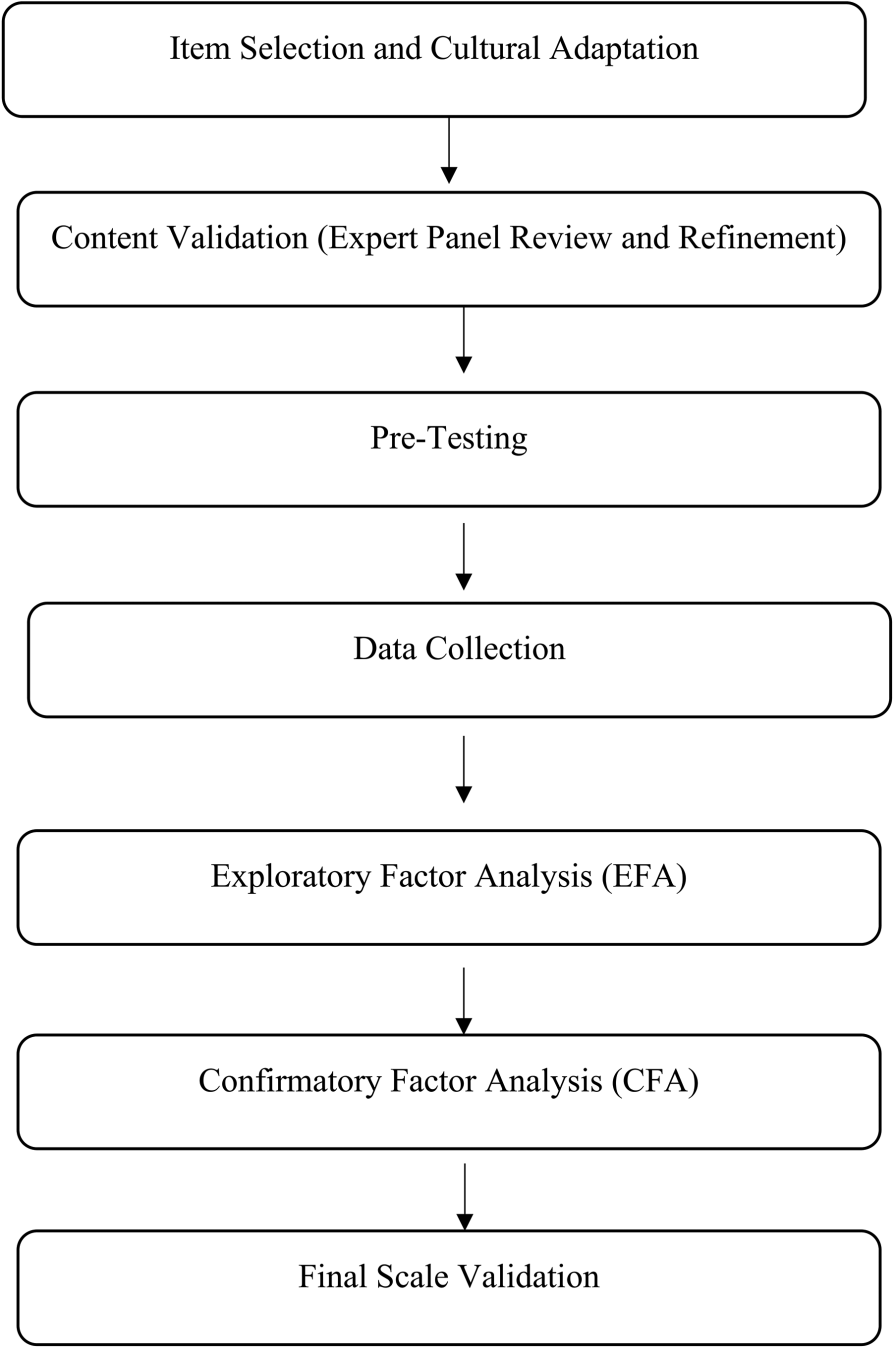
Flowchart of the scale adaptation and validation process for HFK_PS and HFP_PS.

The scale adaptation and validation of the HFK_PS and HFP_PS scales followed key steps: Selection of Items and Cultural Adaptation; Content validation and refining items with expert review; Pre-testing entailed verifying comprehension among preschoolers. An exploratory factor analysis (EFA) was employed to identify the factor structures. A confirmatory factor analysis (CFA) was subsequently employed to ensure the validity of the results Figure 1.

## Design

The cross-sectional study involved a random sample of preschool children and was conducted in Harar City, the capital of the Harari people's region, situated 524 kilometers east of Addis Ababa, Ethiopia. Data was collected from June 1 to 25, 2023.

## Sample size and study population

Scale adaptation studies recommend that the sample size should consist of five to twenty times as many individuals as the total number of items in the scale (22, 23). Tabachnick and Fidell recommend having at least 300 samples for factor analysis (24). Since our study included 9 items, our final sample of 319 preschoolers resulted in approximately 35 participants per item. Thus, 319 preschool children were selected from five randomly chosen kindergartens in the Harari region: two public schools (Jegnoch and Menfesawi) and three private schools (Mekane Sellasie, Hi_Tech, and Hohte Misrak). To ensure a representative sample based on key factors such as socioeconomic status, we randomly selected these schools from a total of four public and 53 private kindergartens. Participants were recruited using systematic random sampling, selecting every kth child from the student roster based on the total enrollment size of each school divided by the required sample size. According to the Harari Region Education Bureau, 53 private kindergartens in the region enrolled 9,016 students, while four public kindergarten schools enrolled approximately 300 students (25). Children were eligible to participate in the study if they were aged 3–7 years and had written informed consent from their parents. The consent form was distributed to all students via their homework folders. The principal investigator and other members of the research team were available to address any questions during child drop-off and pick-up times. Written and signed consent was obtained from parents who expressed willingness to participate in the study.

## Data collection tools

The data collection employed the “Healthy Food Knowledge of Preschool Children (HFK_PS)” and “Healthy Food Preference of Preschool Children (HFP_PS)”.

## The HFK_Ps and HFP_Ps scale

This scale was developed by adapting a known scale, the iPad activity, to measure preschool children's knowledge and preferences related to food and physical activity (14). The iPad activity featured 10 pairs of food photos: 'Sultanas vs. Mixed Lollies,' “Rice vs. Chips”, “Yoghurt vs. Doughnuts”, “Salad Sandwich vs. Jam Sandwich”, “Water vs. Juice”, “Oranges vs. Cookies”, “Rice Cakes vs. Crisps”, “Weetbix vs. Frootloops”, “Milk vs. Cola”, and “Apples vs. Cake”.

To tailor the tool for eastern Ethiopia, we replaced the food images with locally representative options, taking into account regional food culture and availability. Consequently, ten of the foods, such as Rice cakes, Weetbix, Sultanas, Mixed lollies, Frootloops, crisps, Salad Sandwich, Jam Sandwich, Apples, and Oranges, were excluded due to unavailability/minimum consumption in the study area. In their stead, Kinche, Banana, Macaroni, Injera, Mirinda, Atmit, Fanta, and Lollipop were incorporated.

The modified questionnaire included 9 pairs of food images, each presenting one option as a healthy choice and the other as a relatively less healthy or unhealthy alternative. The array of healthy choices comprised Kinche, Banana, Macaroni, Rice, Injera, Milk, Water, Atmit, and Yogurt. Conversely, less healthy or unhealthy options included Cake, Lollipop, Doughnut, Chips, Cookies, Cola, Canned juice, Mirinda, and Fanta. Further details on Healthy Food Knowledge and Preferences are provided in Data Sheet 1 (Supplementary File).

## Data collection and measurement

Pediatric nurses presented pairs of photos to collect data from preschool children. In their respective preschool center, each child participated individually, and the entire process took approximately 7–10 min per child. Pediatric nurses were chosen because of their familiarity with child developmental stages and nutritional needs, enabling them to engage with preschool children during the photo-based activity effectively. Their role was strictly limited to data collection under close supervision to ensure consistency and reliability across all study sites. To assess their knowledge of healthy foods, each child was provided with a doll and instructed to identify the foods that would promote the doll's well-being. We employed dolls to enhance the assessment's interactivity and engagement, acknowledging that young children respond more favorably to play-based approaches than to direct inquiries. Framing the task as “choosing food to keep the doll healthy” made it more relatable, allowing children to naturally demonstrate their understanding of healthy foods. This approach also reduced response bias, as children made choices for the doll rather than feeling pressured to be “correct”. Prior research supports this method, showing that dolls enhance engagement, cognitive accessibility, and accurate expression of dietary knowledge while reducing random guessing (14). On the other hand, during the healthy food preference assessment, children directly stated their preferred foods without utilizing the doll, ensuring that their choices reflected personal preferences rather than what they thought was “best for the doll”. Scoring involved assigning one point for a healthy choice and zero points for a less healthy or unhealthy choice. Furthermore, the child's socio-demographic data were gathered.

To ensure objectivity and consistency, we implemented several measures. Pediatric nurses, trained in standardized data collection, conducted assessments to minimize interviewer bias. These nurses were unaffiliated with the participating kindergartens, ensuring neutrality. The training emphasized uniform administration, avoiding leading questions, and maintaining a neutral demeanor. Data collection took place in a controlled, distraction-free environment, with individual assessments to prevent peer influence and ensure responses reflected each child's knowledge and preferences.

## Operational definition

Kinche, a staple in Ethiopian cuisine, is made with cracked wheat, spiced butter or oil, salt, and optional additions such as onions and tomatoes, offering a versatile option enjoyed both for breakfast and as a component in various meals.

Injera is a pancake-like, soft, sour, circular flatbread, and a staple food for the majority of Ethiopians, made from varieties of tef grain (Eragrostis tef) (26).

Atmit is the Ethiopian version of oatmeal cream that resembles a light, filling porridge, widely consumed as a comforting drink by both youngsters and adults.

The extent to which a scale measures its intended construct defines its validity. Content validity pertains to how well a measure or scale has appropriately represented the content domain it was intended to assess. Testing a scale against theoretically derived hypotheses about the characteristics of the underlying variable or construct is known as construct validity (27). Content validity was assessed by evaluating each item based on criteria such as “essential”, “useful but not essential”, and “not necessary”. For this evaluation, a panel of 5–10 experts is generally considered sufficient (28). Construct validity was measured by Exploratory Factor Analysis (EFA) and Confirmatory Factor Analysis (CFA).

## Data analysis

IBM SPSS Statistics version 25 was used for EFA, whereas LISREL version 8.8 was used for CFA. Validity testing involved the assessment of both content validity and construct validity.

The evaluation of content validity underwent two phases. Initially, a panel consisting of 10 experts—comprising six pediatric nurses, two public health nutritionists, and two kindergarten teachers—was assigned the task of rating a 13-item scale on a 1–3 scale (3 for “essential”, 2 for “useful but not essential”, and 1 for “not necessary”). Following this assessment, three items underwent modifications, and four items were eliminated based on expert opinions. Reasons for exclusion included factors such as unavailability or limited consumption in the study area and redundancy in item conceptualization.

In the second phase, the revised set of 9 items underwent evaluation by the same panel of 10 experts. During this stage, experts were instructed to rate the tool on a 1–4 scale (1 = very little change required, 2 = little change required, 3 = appropriate, and 4 = very convenient) to assess the appropriateness of each item in the scale (29). Notably, none of the experts assigned 1 or 2 points to any item, indicating a lack of recommended changes. Individual content validity indices (I-CVI) were computed for each item, ranging from 0.9 to 1.0, while the scale-level content validity index (S-CVI) was determined to be 0.98, illustrating consistency in the evaluation process. A pre-test study was conducted with 19 preschool children from schools other than those included in the main study to assess the comprehensibility and feasibility of the data collection process. This phase evaluated children's ability to distinguish between healthy and less healthy foods, the cultural relevance of the modified food images, the time required per child to complete the activity, and any challenges in administering the tool and scoring responses. The findings indicated that no major modifications were necessary, as children successfully engaged with the visual stimuli and understood the task as intended. However, minor refinements were made to streamline the interview process and enhance the clarity of instructions.

Construct validity was examined through EFA and CFA. Before conducting EFA, we evaluated the data fit by calculating the Kaiser-Meyer-Olkin (KMO) measure to assess sampling adequacy. Additionally, Bartlett's Test of Sphericity was employed to determine the suitability of the data for factor analysis (24). The EFA was conducted with principal component analysis, and direct oblimin rotation with Kaiser Normalization. As a factor extraction metric, we looked at the Eigen value of greater than 1.0 and scree plot (30).

In conducting confirmatory factor analysis, various fit indices were calculated to assess the model's goodness of fit. These included the ratio of chi-square to degree of freedom (*χ*^2^/df), Root Mean Square Error of Approximation (RMSEA), Root Mean Square Residual (RMR), Standardized RMR, Normed Fit Index (NFI), Non-Normed Fit Index (NCFI), Comparative Fit Index (CFI), Incremental Fit Index (IFI), Relative Fit Index (RFI), Goodness of Fit Index (GFI), and Adjusted Goodness of Fit Index (AGFI). Criteria for an acceptable model fit were set as follows: *χ*^2^/df less than 3.0; a *χ*^2^ *p*-value less than 0.05; RMSEA less than 0.08; NFI, NNFI, CFI, IFI, RFI, GFI, and AGFI values falling above 0.90 (31–33). The Cronbach's alpha reliability coefficient was computed to evaluate the scale's reliability. An *α* value of 0.80 or higher is considered good, 0.60–0.70 is considered acceptable, and less than 0.60 is considered poor (34).

Group differences in the HFK_PS and HFP_PS were analyzed using independent samples t-tests for gender comparisons and one-way ANOVA for age and grade-level variations. However, Levene's test indicated significant variance heterogeneity in age-based comparisons of food knowledge, violating ANOVA's equal variance assumption (24). To address this, Welch's ANOVA was applied, ensuring robust and unbiased comparisons by adjusting for variance heterogeneity (35, 36). Effect sizes (partial eta squared, *η*²*p*) were reported to quantify group differences (37), and Tukey's HSD *post-hoc* tests identified significant pairwise differences (38). Statistical significance was set at *p* < 0.05, ensuring rigor in hypothesis testing.

## Ethical consideration

This study was conducted according to the guidelines laid down in the Declaration of Helsinki. Ethical approval was obtained from the Haramaya University Institutional Health Research and Ethics Review Committee with reference number IHRERC/068/2023 on 18 April 2023. Written and informed consent was obtained from all the parents in the study. All collected data were anonymized by assigning unique identification codes instead of using personal identifiers. Only authorized members of the research team had access to the dataset, and data were stored securely on a password-protected system to prevent unauthorized access. Additionally, during analysis, no individual responses were linked to identifiable information, ensuring complete anonymity. The study adhered to institutional and ethical guidelines, and all findings were presented in an aggregated format.

## Results

### Participants' characteristics

The study included 319 preschool children, with a nearly equal gender distribution of 165 boys (51.7%) and 154 girls (48.3%). The mean age of the children was 5.45 ± 0.75 years, ranging from 3 to 7 years. The majority were 6–7 years old (59.2%), followed by 5–6 years (28.2%), 4–5 years (11.0%), and a small fraction of 3–4-year-olds (1.6%). In terms of grade levels, 21.3% were in kindergarten 1, 47.6% in Kindergarten 2, and 31.0% in Kindergarten 3. Notably, 91.8% of the children watched television once a week, while 8.2% did not. The sample included children from both private (68.0%) and government (32.0%) schools, with the largest group attending Mekan Silasie (39.2%), followed by Jegnoch (32.0%), Hi_tech Nok (16.3%), and Hohte Misrak (12.5%) Table 1.

**Table 1:** Socio-demographic characteristics of study participants.

Characteristics	Categories	Frequencies	Percentages
Sex	Boys	165	51.70
	Girls	154	48.30
Age in years	3–4	5	1.60
	4–5	35	11.00
	5–6	90	28.20
	6–7	189	59.20
Grade	Kindergarten 1	68	21.30
	Kindergarten 2	152	47.60
	Kindergarten 3	99	31.00
Watch television once a week	Yes	293	91.80
	No	26	8.20
Type of school	Private	217	68.00
	Government	102	32.00
Name of schools	Mekan Silasie	125	39.20
	Jegnoch	102	32.00
	Hi_tech Nok	52	16.30
	Hohte Misrak	40	12.50

## Validity of the HFK_Ps and HFP_Ps

### Explanatory factor analysis

A KMO measure of 0.84 was obtained for HFK_PS and 0.82 for HFP_PS. Bartlett's sphericity test yielded chi-square values of 668.89 for HFK_PS and 605.57 for HFP_PS, with df = 36 (*P* < 0.000). This confirms that the sample is suitable and adequate for factor analysis. The EFA indicated a two-factor construct for HFK_PS that explained 50.91% of the total variance, with eigenvalues of 3.53 and 1.05, respectively. Factor 1, representing “foods that are edible items”, accounted for 39.21% of the variance, and Factor 2, representing “fluids that include drinks/beverages”, accounted for 11.70%. Similarly, for HFP_PS, the EFA also showed a two-factor construct with eigenvalues of 3.12 and 1.20, explaining 50.18% of the total variance. Factor 1 contributed 36.80%, and Factor 2 contributed 13.38% to the variance.

Table 2 presents the factor loadings for the Healthy Food Knowledge and Preferences of Preschool Children (HFK_PS) scale, derived from both Exploratory Factor Analysis (EFA) and Confirmatory Factor Analysis (CFA). Each of the nine items (Items 1–9) showed high component loadings in both scales (39). In the foods category, the items display strong factor loadings in the EFA, ranging from 0.45 to 0.79. Specifically, the item “Kinche vs. Cake” (FK1) has a high loading of 0.79, indicating a strong relationship with the food factor. Other notable loadings include “Banana vs. Lollipop” (FK2) at 0.77, “Doughnuts vs. Macaroni” (FK3) at 0.70, and “Rice vs. Chips” (FK4) at 0.60. The item “Enjera vs. Cookies” (FK5) shows the lowest loading within the foods category at 0.45, likely due to the high familiarity and frequent consumption of both Injera and Cookies, reducing discrimination. Preschoolers may not have perceived a strong health difference. The CFA results for the foods category confirm the structure identified in the EFA, with loadings ranging from 0.51 to 0.71. “Rice vs. Chips” (FK4) has the highest loading at 0.71, followed by “Doughnuts vs. Macaroni” (FK3) at 0.67 and “Banana vs. Lollipop” (FK2) at 0.65. The item “Kinche vs. Cake” (FK1) has a loading of 0.51, and “Enjera vs. Cookies” (FK5) shows an improved loading of 0.55 compared to the EFA.

**Table 2:** Factor loadings for healthy food knowledge and preferences of preschool children.

Item code	Item	Healthy food knowledge				Healthy food preferences			
		EFA, factor loading		CFA, factor loading		EFA, factor loading		CFA, factor loading	
		Foods	Fluids	Foods	Fluids	Foods	Fluids	Foods	Fluids
FK1	Kinche vs. Cake	0.79		0.51		0.75		0.65	
FK2	Banana vs. lollipop	0.77		0.65		0.69		0.52	
FK3	Doughnuts vs. Macaroni	0.70		0.67		0.68		0.63	
FK4	Rice vs. Chips	0.60		0.71		0.65		0.58	
FK5	Enjera vs. cookies	0.45		0.55		0.57		0.44	
FK6	Milk vs. Cola		0.80		0.47		0.91		0.75
FK7	Water vs. Canned juice		0.75		0.64		0.77		0.67
FK8	Mirinda vs. Atmit		0.60		0.65		0.71		0.62
FK9	Fanta vs. Yogurt		0.49		0.54		0.44		0.50

In the fluids category, the EFA results indicate strong loadings, with “Milk vs. Cola” (FK6) having the highest loading at 0.80, followed by “Water vs. Canned Juice” (FK7) at 0.75 and “Mirinda vs. Atmit” (FK8) at 0.60. “Fanta vs. Yogurt” (FK9) has the lowest loading within this category at 0.49. The CFA results for the fluids category reveal a range of factor loadings from 0.47 to 0.65, supporting the factor structure identified in the EFA. “Water vs. Canned Juice” (FK7) and “Mirinda vs. Atmit” (FK8) both have loadings of 0.65, indicating strong relationships with the fluids factor. “Banana vs. Lollipop” (FK2) and “Fanta vs. Yogurt” (FK9) show loadings of 0.64 and 0.54, respectively. The item “Milk vs. Cola” (FK6) has a loading of 0.47, which is slightly lower than its EFA counterpart but still significant Table 2.

The EFA results for the Healthy Food Preferences of Preschool Children (HFP_PS) scale in the foods category show also strong factor loadings, ranging from 0.57 to 0.75. The item “Kinche vs. Cake” (FP1) has the highest loading at 0.75, suggesting a strong association with the food preference factor. Other significant loadings include “Banana vs. Lollipop” (FP2) at 0.69, “Doughnuts vs. Macaroni” (FP3) at 0.68, and “Rice vs. Chips” (FP4) at 0.65. The item “Enjera vs. Cookies” (FP5) shows the lowest loading in this category at 0.57. The CFA results for the foods category corroborate the EFA findings, with loadings ranging from 0.44 to 0.65. “Kinche vs. Cake” (FP1) has a high loading of 0.65, followed by “Doughnuts vs. Macaroni” (FP3) at 0.63 and “Rice vs. Chips” (FP4) at 0.58. The item “Banana vs. Lollipop” (FP2) shows a loading of 0.52, while “Enjera vs. Cookies” (FP5) has a lower loading of 0.44 compared to the EFA.

In the fluids category, the EFA results indicate strong factor loadings, with “Milk vs. Cola” (FP6) having the highest loading at 0.91. Other notable loadings include “Water vs. Canned Juice” (FP7) at 0.77, “Mirinda vs. Atmit” (FP8) at 0.71, and “Fanta vs. Yogurt” (FP9) at 0.44. The CFA results for the fluids category support the EFA structure, with loadings ranging from 0.50 to 0.75. “Milk vs. Cola” (FP6) has the highest loading at 0.75, followed by “Water vs. Canned Juice” (FP7) at 0.67 and “Mirinda vs. Atmit” (FP8) at 0.62. The item “Fanta vs. Yogurt” (FP9) shows a loading of 0.50.

### Confirmatory factor analysis

**Figure 2:**
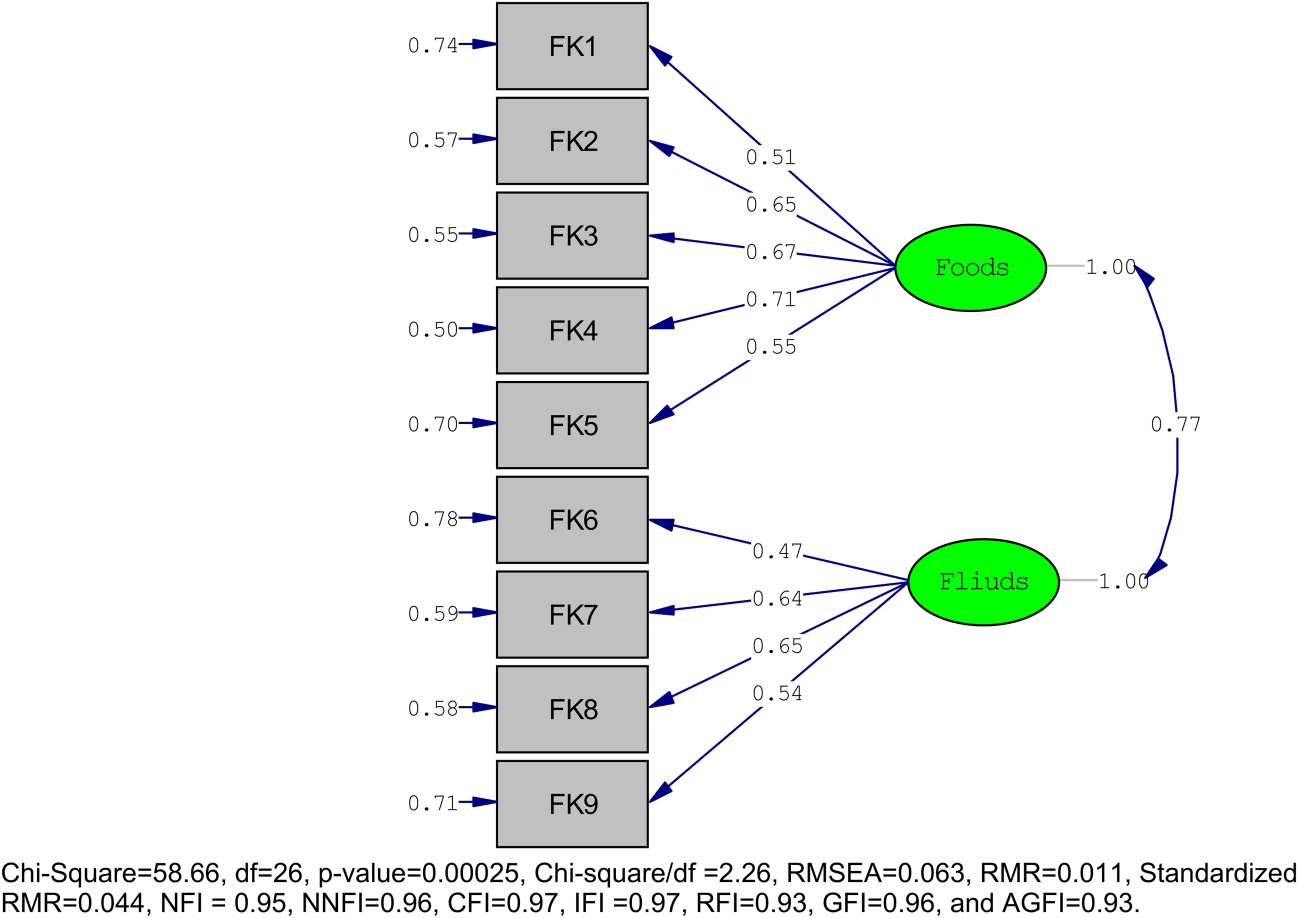
Confirmatory factor analysis with model fitness of HFK_PS.

The two-factor model derived from the EFA was fitted using the CFA for both HFK_PS and HFP_PS. Jöreskog and Sörbom's criteria were satisfied because there were no red arrows in the *t*-values, indicating that there were no problematic items on the scale (40). For HFK_PS, all factor loadings exceed the recommended threshold of 0.30, ranging from 0.47 to 0.71. A *χ*^2^/df value of 2.26 and a *χ*^2^ *p*-value below 0.05 further suggest a robust fit, according to the model fit analysis. The model's overall sound fit was confirmed by the RMSEA value of 0.063 and all metrics, including NFI, NNFI, CFI, IFI, RFI, GFI, and AGFI, surpassing the 0.90 threshold Figure 2.

**Figure 3:**
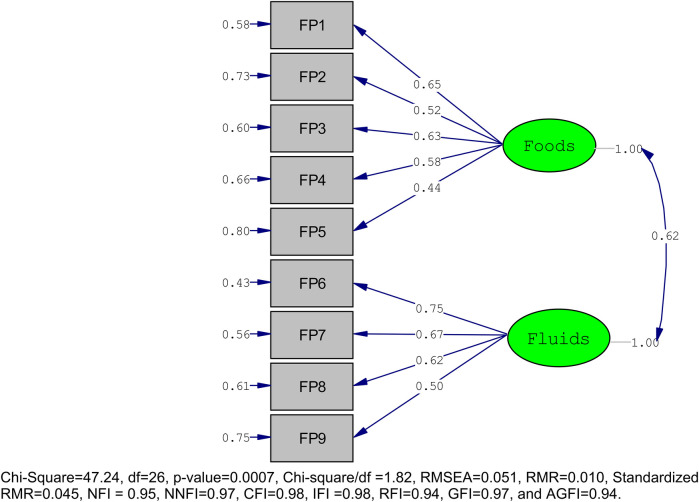
Confirmatory factor analysis with model fitness of HFP_PS.

Similarly, for HFP_PS, the two-factor solution had an acceptable fit to the data with *χ*^2^/df = 1.82, RMSEA = 0.051, and all metrics, including NFI, NNFI, CFI, IFI, RFI, GFI, and AGFI, surpassing the 0.90 threshold Figure 3.

### Reliability

The Cronbach's *α* values for internal consistency were 0.80, 0.76, and 0.66 for the HFK_PS total score, Foods (factor 1), and Fluids (factor 2), respectively. This indicates the scale's good internal consistency. The item-total statistics for the HFK_PS indicated moderate to strong relationships between items and the total scale score, with corrected item-total correlations ranging from 0.38 to 0.60. The Cronbach's alpha values if items are deleted range from 0.77 to 0.80, suggesting that the internal consistency of the scale would not significantly improve with the removal of any single item. Overall, the statistics confirm that all items contribute positively to the internal consistency of the HFK_PS scale, demonstrating good reliability for both the total scale and its sub-factors Table 3.

**Table 3:** Item-total statistics of HFK_PS and HFP_PS scale.

Item code	Item	Healthy food knowledge (HFK_PS) scale			Healthy food preferences (HFP_PS) scale		
		Scale mean if item deleted	Corrected item-total correlation	Cronbach's alpha if item deleted	Scale mean if item deleted	Corrected item-total correlation	Cronbach's alpha if item deleted
FK1	Kinche vs. Cake	3.78	0.42	0.79	2.85	0.52	0.76
FK2	Banana vs. Lollipop	3.97	0.53	0.78	2.86	0.41	0.77
FK3	Doughnuts vs. Macaroni	3.99	0.57	0.78	2.88	0.50	0.76
FK4	Rice vs. Chips	3.91	0.60	0.77	2.89	0.47	0.76
FK5	Enjera vs. cookies	4.01	0.49	0.79	2.87	0.36	0.78
FK6	Milk vs. Cola	3.74	0.38	0.80	2.83	0.51	0.76
FK7	Water vs. Canned juice	3.82	0.51	0.78	2.77	0.49	0.76
FK8	Mirinda vs. Atmit	3.95	0.51	0.78	2.81	0.49	0.76
FK9	Fanta vs. Yogurt	3.88	0.46	0.79	2.80	0.47	0.76

The HFP_PS total score and its constructs, Foods (factor 1) and Fluids (factor 2), exhibited good internal consistency with Cronbach's α values of 0.78, 0.70, and 0.72, respectively. The item-total statistics for the Healthy Food Preference Scale (HFP_PS) showed corrected item-total correlations ranging from 0.36 to 0.52, indicating moderate relationships between individual items and the total scale score. For instance, item FP1 (“Kinche vs. Cake”) has a strong correlation (0.52) and a Cronbach's alpha if deleted of 0.76, while item FP5 (“Enjera vs. Cookies”) has the lowest correlation (0.36) and a Cronbach's alpha if deleted of 0.78. Overall, Cronbach's alpha values range from 0.76 to 0.78, suggesting that the internal consistency of the scale would not significantly improve with the removal of any single item. These results confirm that all items contribute positively to the internal consistency of the HFP_PS scale, demonstrating good reliability.

### Effect of age on healthy food knowledge and healthy food preference

An analysis of variance (ANOVA) was conducted to compare the effects of age on healthy food knowledge and healthy food preference among preschool children. For healthy food knowledge, the results revealed a significant effect of age, F (3, 19.46) = 7.67, *p* = .001, *η*²*p* = .05. In contrast, the ANOVA did not show a significant effect of age on healthy food preference, F(3, 315) = 0.15, *p* = 0.928, *η*²*p* = 0.001. These results show that while healthy food knowledge increases significantly with age, healthy food preferences do not show significant variation across the age groups Table 4.

**Table 4:** Effect of age on healthy food knowledge and healthy food preference.

Variable	Age	M	SD	SE	95% CI of mean		F (df)	Sum of squares	*p*-value	*η*²*p*
Healthy food knowledge	3–4 years (*N* = 5)	3.40	1.67	0.75	1.32	5.48	F (3,19.46) * = 7.67	108.68	0.001	0.045
	4–5 years (*N* = 35)	2.97	1.82	0.31	2.35	3.60				
	5–6 years (*N* = 90)	4.16	2.66	0.28	3.60	4.71				
	6–7 years (*N* = 189)	4.78	2.89	0.21	4.36	5.19				
Healthy food Preference	3–4 years (*N* = 5)	3.20	2.17	0.97	0.51	5.89	F (3,315) = 0.15	3.11	0.928	0.001
	4–5 years (*N* = 35)	3.09	2.13	0.36	2.35	3.82				
	5–6 years (*N* = 90)	3.07	2.49	0.26	2.54	3.59				
	6–7 years (*N* = 189)	3.28	2.75	0.20	2.88	3.67				

Welch homogeneity correction.

*Post hoc* comparisons using the Tukey HSD test indicated a significant mean difference in healthy food knowledge between the 4–5 year and 6–7-year groups, MD = −1.81, SE = 0.50, *p* = .002, 95% CI [−3.10, −0.52]. This suggests that children in the 6–7-year age group have significantly higher healthy food knowledge compared to those in the 4–5-year age group Table 5.

**Table 5:** *Post-hoc* tests (multiple comparison) of healthy food knowledge score among age groups.

Age		Mean difference	SE	*t*	*p*-value (Tukey)
(3–4 years)	(4–5 years)	0.43	1.30	0.33	0.99
	(5–6 years)	−0.76	1.25	−0.61	0.93
	(6–7 years)	−1.38	1.23	−1.12	0.68
(4–5 years)	(5–6 years)	−1.18	0.54	−2.19	0.13
	(6–7 years)	−1.81	0.50	−3.62	0.00
(5–6 years)	(6–7 years)	−0.62	0.35	−1.79	0.28

**Figure 4:**
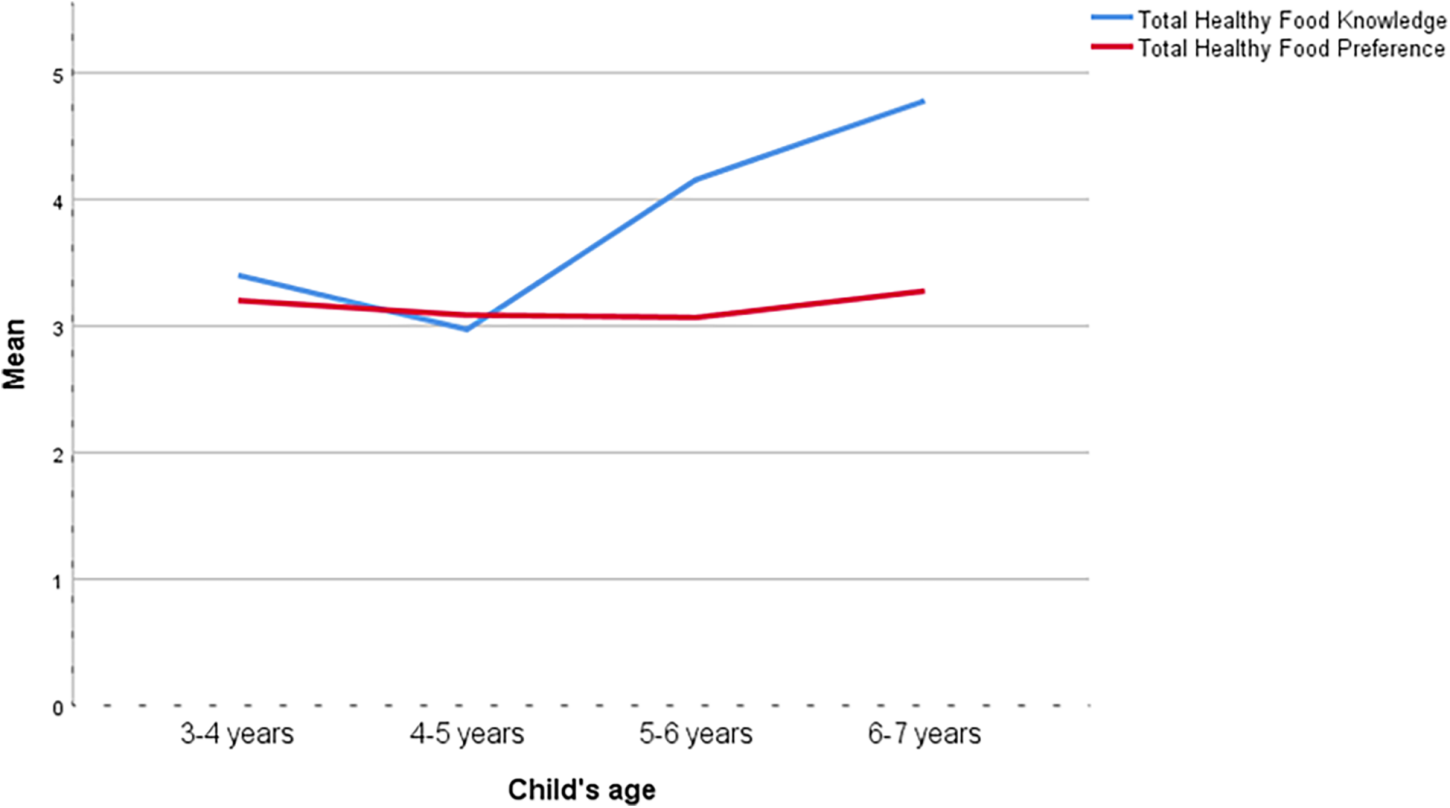
Mean scores of healthy food knowledge and healthy food preference by child's age.

In Figure 4, the blue line, representing healthy food knowledge, shows a significant increase with age. This trend indicates that older children have greater knowledge of healthy foods. In contrast, the red line, representing healthy food preference, remains relatively stable across age groups, suggesting that children's preferences for healthy foods do not significantly change with age Figure 4.

### Effect of grade level on healthy food knowledge and healthy food preference

The ANOVA examined the effect of grade level on Healthy Food Knowledge and Healthy Food Preference among preschool children. The results indicated significant differences across the three grade levels (KG-1, KG-2, KG-3) for both variables. For Healthy Food Knowledge, mean scores increased from KG-1 (M = 3.15, SD = 1.86) to KG-2 (M = 4.11, SD = 2.79) and KG-3 (M = 5.65, SD = 2.76), with an ANOVA showing a significant effect, F(2, 316) = 20.01, *p* < 0.001, *η*²*p* = 0.112. Similarly, for Healthy Food Preference, mean scores increased from KG-1 (M = 2.93, SD = 1.86) to KG-2 (M = 2.95, SD = 2.63) and KG-3 (M = 3.75, SD = 2.91), with a significant effect found, F(2, 316) = 24.28, *p* < 0.001, *η*²*p* = 0.112. These findings suggest that both Healthy Food Knowledge and Preference improve as children advance in grade level Table 6.

**Table 6:** Effect of grade level on healthy food knowledge and healthy food preferenc*e.*

Variable	Age	M	SD	SE	95% CI of mean		F (df)	Sum of squares	*p*-value	*η*²*p*
Healthy food knowledge	KG-1 (*N* = 68)	3.15	1.86	0.23	2.70	3.60	F (2, 316) = 20.01	136.54	<0.001	0.112
	KG-2 (*N* = 152)	4.11	2.79	0.23	3.66	4.56				
	KG-3 (*N* = 99)	5.65	2.76	0.28	5.10	6.20				
Healthy food Preference	KG-1 (*N* = 68)	2.93	1.86	0.23	2.48	3.38	F (2,316) = 24.28	136.54	<0.001	0.112
	KG-2 (*N* = 152)	2.95	2.63	0.21	2.53	3.38				
	KG-3 (*N* = 99)	3.75	2.91	0.29	3.17	4.33				

The *post-hoc* tests (Tukey) for Healthy Food Knowledge and Healthy Food Preference among different grade levels revealed significant differences. For Healthy Food Knowledge, the comparison between KG-1 and KG-2 showed a mean difference of −0.97 (SE = 0.38, *t* = −2.53, *p* = 0.032), indicating that KG-2 children had significantly higher knowledge scores than KG-1 children. The comparison between KG-1 and KG-3 showed a mean difference of −2.50 (SE = 0.41, *t* = −6.08, *p* < .001), with KG-3 children having significantly higher knowledge scores than KG-1 children. Additionally, the comparison between KG-2 and KG-3 showed a mean difference of −1.54 (SE = 0.34, *t* = −4.55, *p* < .001), indicating that KG-3 children had significantly higher knowledge scores than KG-2 children. For Healthy Food Preference, the *post-hoc* tests showed one significant difference. The comparison between KG-2 and KG-3 revealed a mean difference of −0.79 (SE = 0.33), *t* = −2.38, *p* = 0.047), indicating that KG-3 children had a significantly higher preference for healthy food than KG-2 children. Other comparisons did not show statistically significant differences Table 7.

**Table 7:** *Post-hoc* tests (multiple comparison) of healthy food knowledge score among grade levels.

Variable	Grade level		Mean difference	SE	*t*	*p*-value (Tukey)
Healthy Food Knowledge	KG-1	KG-2	−0.97	0.38	−2.53	0.032
		KG-3	−2.50	0.41	−6.08	<.001
	KG-2	KG-3	−1.54	0.34	−4.55	<.001
Healthy Food Preference	KG-1	KG-2	−0.03	0.38	−0.07	0.997
		KG-3	−0.82	0.41	−2.02	0.109
	KG-2	KG-3	−0.79	0.33	−2.38	0.047

### Effect of gender on healthy food knowledge and healthy food preference

Table 8 presents the results of an independent sample t-test comparing Healthy Food Knowledge and Healthy Food Preference between male and female preschool children. The mean score for Healthy Food Knowledge was 4.26 (SD = 2.75) for males (*N* = 165) and 4.51 (SD = 2.78) for females (*N* = 154), with no significant difference found between the genders, t(317) = −0.82, *p* = 0.416, and a mean difference of −0.25 (95% CI [−0.86, 0.36). Similarly, the mean score for Healthy Food Preference was 3.17 (SD = 2.54) for boys and 3.22 (SD = 2.68) for girls, also showing no significant difference, t(317) = −0.18, *p* = 0.861, and a mean difference of −0.05 (95% CI [−0.63, 0.52). These findings indicate that there are no significant differences in Healthy Food Knowledge and Healthy Food Preference between male and female preschool children Table 8.

**Table 8:** Independent sample *t*-test of gender with healthy food knowledge and healthy food preference of preschool children.

Variable	Gender				*t*(df)	*p*-value	Mean difference	95% CI for mean difference	
	Male (*N* = 165)		Female (*N* = 154)						
	Mean	*SD*	Mean	*SD*				Lower	Upper
Healthy Food Knowledge	4.26	2.75	4.51	2.78	t (317) = −0.82	0.416	−0.25	−0.86	0.36
Healthy Food Preference	3.17	2.54	3.22	2.68	t (317) = −0.18	0.861	−0.05	−0.63	0.52

### Correlation between health food knowledge and healthy preferences scores

There is a strong positive correlation between “Total Food Knowledge” and “Total Food Preference” (*r* = .43, *p* < .001), suggesting that higher overall food knowledge is associated with greater preference for healthy foods. Similarly, individual food knowledge variables (FK1-FK9) generally show significant positive correlations with “Total Food Knowledge”, with FK4 (Rice vs. Chips) having the highest correlation (*r* = .71, *p* < .001).

Food preference variables (FP1-FP9) are also positively correlated with “Total Food Preference”, with FP1 (*r* = .65, *p* < .001) and FP6 (*r* = .64, *p* < .001) showing the strongest relations. This indicates that children's preferences for specific healthy foods contribute significantly to their overall healthy food preferences. Moreover, several individual food knowledge variables show significant correlations with specific food preferences. For instance, FK1 (Kinche vs. Cake) has a significant positive correlation with FP2 (*r* = .13, *p* < .05) and FP5 (*r* = .15, *p* < .01), indicating that knowledge in these areas may influence children's preferences for these specific foods Table 9.

**Table 9:** Correlation between health food knowledge and healthy preferences scores.

Items	FP1	FP2	FP3	FP4	FP5	FP6	FP7	FP8	FP9	Total FK	Total FP
FK1	0.02	.13*	0.08	0.11	.15**	0.04	0.06	0.08	−0.09	.56***	0.1
FK2	.15**	.29***	.29***	.23***	0.11	.16**	.14*	.17**	.12*	.65***	.30***
FK3	.17**	.28***	.16**	.24***	.16**	.16**	.14*	.19***	.15**	.68***	.30***
FK4	.16**	.24***	.20***	.29***	.22***	.18**	.17**	.23***	0.11	.71***	.33***
FK5	0.1	.27***	.23***	.26***	.14*	.16**	.13*	.17**	.15**	.62***	.29***
FK6	0.11	.19***	.16**	.21***	0.08	.16**	.22***	.20***	.21***	.52***	.28***
FK7	.16**	.18**	.24***	.15**	0.1	.26***	.28***	.23***	0.09	.64***	.31***
FK8	0.09	.16**	.13*	.20***	0.11	.22***	.26***	.27***	.16**	.63***	.30***
FK9	0.05	.12*	0.04	.19***	.14*	.17**	.14*	.20***	0.09	.59***	.21***
Total FK	.18**	.33***	.27***	.33***	.22***	.27***	.27***	.31***	.30***	—	.43***
Total FP	.65***	.55***	.63***	.60***	.51***	.64***	.63***	.63***	.61***	.43***	—

* *P*-value less than 0.05, **P-value less than 0.01, ***P-value less than 0.001.

## Discussion

We developed the HFK_PS and HFP_PS scales using a direct, child-centered approach to assess preschool children's knowledge and preferences regarding healthy foods. These scales demonstrated strong internal consistency and appropriate factor structures, indicating proper validity and reliability. These align with a previous study that validated a food knowledge and preference assessment tool tailored for young children, particularly those incorporating culturally adapted methodologies and visual-based evaluations (14).

The construct validity of the Healthy Food Knowledge (HFK_PS) and Healthy Food Preference (HFP_PS) scales was confirmed using Exploratory Factor Analysis (EFA) and Confirmatory Factor Analysis (CFA). The EFA identified a two-factor structure for both scales, explaining an acceptable level of variance, while CFA demonstrated a good model fit (e.g., RMSEA < 0.08, CFI > 0.90). These findings are consistent with previous research that has validated similar food knowledge and preference assessment tools (14). Additionally, the statistical thresholds used for validity assessment align with recommendations in psychometric literature (24, 27), further supporting the robustness of our results.

The Healthy Food Knowledge (HFK_PS) and Healthy Food Preference (HFP_PS) scales used a photo-based assessment to evaluate preschool children's understanding and preferences for healthy foods. A study that adapted visual tools for food knowledge and preference assessment in different populations validated this method (14). The effectiveness of this method is supported by developmental psychology research, which shows that preschool-aged children respond more accurately to image-based assessments than text-based surveys (21, 41).

The content validity of the HFK_PS and HFP_PS scales was established through a structured expert panel review, ensuring the scales appropriately measure healthy food knowledge and preferences among preschool-aged children. Similar validation approaches have been employed in studies adapting nutrition-related assessment tools for diverse populations, confirming the effectiveness of expert evaluations in refining scale items (19, 21). The high Scale-level Content Validity Index (S-CVI = 0.98) observed in this study aligns with findings from other culturally adapted food knowledge and preference scales, reinforcing the robustness of the validation process. Such rigorous expert assessment methods are widely recognized in psychometric research as essential for ensuring content validity (28).

Furthermore, the Healthy Food Knowledge Activity (HFKA) and the Food Preference Questionnaire (FPQ) also demonstrated strong content validity through extensive expert evaluations. The HFKA uses photo-based tools to engage young children and assess their ability to classify foods as healthy or unhealthy, similar to our use of culturally relevant images in the HFK_PS to ensure developmental appropriateness and engagement (14). Overall, the high content validity achieved in our study aligns well with these established tools, underscoring the robustness of our validation process and the effectiveness of the HFK_PS and HFP_PS scales in assessing healthy food knowledge and preferences among preschool children.

The internal consistency of the HFK_PS and HFP_PS scales falls within the acceptable range for psychometric instruments, as indicated by Cronbach's alpha values of 0.80 and 0.78. These values for reliability are the same as those found in studies that tested similar nutrition-related assessment tools for preschool-aged children in a range of linguistic and cultural settings (14). The reliability coefficients align with standard recommendations in psychometric literature, where α ≥ 0.70 is considered acceptable and α ≥ 0.80 is deemed strong (22, 34). These findings further support the robustness of the adapted HFK_PS and HFP_PS scales in accurately measuring healthy food knowledge and preferences in preschool children.

Similarly, the Healthy Food Knowledge Activity (HFKA), a photo-based tool for assessing nutrition knowledge among children aged 5–6 years, shares methodological similarities with the HFK_PS, as both use visual aids to engage children. However, the HFK_PS's inclusion of culturally relevant food items enhances its applicability and validity for Ethiopian children. The Food Preference Questionnaire (FPQ), which quantifies children's liking or disliking for various foods, offers detailed insights into food preferences. The HFP_PS similarly assesses food preferences but is specifically designed for the Ethiopian context, making it a valuable tool for local nutritional interventions (19, 21).

Our study found that preschoolers' knowledge and preferences about healthy foods were not significantly different between boys and girls. This suggests that external factors like caregivers, schools, and exposure to the wider environment have a bigger effect on both boys and girls than differences that are based on their gender. Previous research has come to a variety of conclusions. Some say that girls are more aware of nutrition because they are socialized with healthier people earlier and their parents encourage them to do healthy things (15, 19). Other studies, especially those with younger children, say there are no significant differences between boys and girls, highlighting the importance of environmental factors over personal gender preferences (42). Our findings align with studies indicating minimal gender differences at the preschool level, reinforcing the need for gender-neutral nutrition interventions to ensure equal opportunities for both boys and girls to develop healthy eating habits. Given that early dietary habits often persist into later life (5), inclusive interventions may be more effective in promoting long-term healthy eating behaviors.

The process of adapting the scales to the Ethiopian context by replacing food items with locally relevant options is consistent with best practices in cross-cultural research. Similar adaptation strategies have been successfully employed in studies from Sri Lanka and Australia, which have emphasized the importance of cultural relevance in dietary assessment tools (19, 21). These adaptations ensure that the tools accurately reflect the dietary habits and preferences of the target population, thereby enhancing the validity of the results. By using a similar methodology, researchers and practitioners in other low- and middle-income countries including in Ethiopia can tailor existing nutrition assessment tools to their local food environments, enhancing the accuracy and effectiveness of early childhood nutrition interventions. Additionally, this adaptation process can be extended to multicultural settings within high-income countries, where dietary habits vary across ethnic groups. Moreover, these tools provide reliable measures for evaluating preschool children's understanding of healthy foods, allowing educators and policymakers to design targeted interventions that promote better dietary habits from an early age. Educators can track progress, identify knowledge gaps, and implement age-appropriate strategies to encourage healthier food choices by incorporating these validated tools into school-based nutrition programs. The results can also help shape public health policies that aim to improve the nutrition of young children, especially in low- and middle-income areas where cultural and socioeconomic factors affect eating habits.

The validated Healthy Food Knowledge (HFK_PS) and Healthy Food Preference (HFP_PS) scales can be adapted for use in other regions or countries by modifying the culturally specific food items while maintaining the overall structure of the tool. Since dietary habits vary across countries, locally available and commonly consumed foods should replace items that may be unfamiliar to children in different settings. The core methodology, including the photo-based interview approach, can remain unchanged, ensuring consistency in assessing food knowledge and preferences. Additionally, the findings of this study have broader implications for low- and middle-income countries (LMICs), where preschool children often face similar challenges related to poor dietary diversity, limited nutrition education, and high consumption of processed foods. The adaptation of such tools across LMICs can support global efforts to improve childhood nutrition, particularly in regions where culturally appropriate assessment instruments are lacking.

The limitations of this study must be acknowledged to provide a balanced understanding of the findings and their implications. One significant limitation is the sample's regional specificity. The study was conducted exclusively in the Harari region of eastern Ethiopia, which may limit the generalizability of the findings to other regions or countries. Cultural, socio-economic, and dietary differences can significantly influence food knowledge and preferences, meaning the scales validated in this study might not be directly applicable to children in different settings without further adaptation and validation. Another limitation is the cross-sectional nature of the study, which only provides a snapshot of the participants' healthy food knowledge and preferences at a single point in time. This design does not allow for the assessment of changes over time or the stability of these constructs. Longitudinal studies would be beneficial to understand how healthy food knowledge and preferences develop and change as children grow, as well as to evaluate the long-term effectiveness of nutrition education interventions.

## Conclusions and recommendations

The present study successfully validated and assessed the reliability of the Healthy Food Knowledge (HFK_PS) and Healthy Food Preference (HFP_PS) scales for preschool children in Harar, Ethiopia. The scales demonstrated strong internal consistency and appropriate factor structures, confirming their suitability for measuring healthy food knowledge and preferences in this context. The results indicated significant improvements in healthy food knowledge and preferences as children advanced in age and grade level, emphasizing the impact of early educational interventions. The significant positive correlation between food knowledge and preferences further suggests that enhancing children's knowledge can lead to healthier food choices.

By integrating these validated tools into early childhood education systems, educators and policymakers can design targeted nutrition interventions that promote healthier eating behaviors from an early age. These scales provide a reliable method for assessing children's understanding of healthy foods, enabling schools to track progress, identify knowledge gaps, and implement effective, age-appropriate nutrition programs. Furthermore, the tools can inform public health policies aimed at improving preschoolers' diets, particularly in low- and middle-income settings where cultural and socioeconomic factors influence eating habits.

Future research should explore the longitudinal impact of using these scales in nutrition education programs, ensuring their long-term effectiveness in shaping children's dietary behaviors. They will also be more reliable and useful in a wider range of cultural and dietary settings if they are tested in different parts of Ethiopia and other low- and middle-income countries. Expanding their use globally will strengthen their role in childhood nutrition research and policy development, ultimately contributing to the reduction of malnutrition and the promotion of lifelong healthy eating habits.

## Data Availability

The original contributions presented in the study are included in the article/Supplementary Material, further inquiries can be directed to the corresponding author.
