# Supplementary material for: Validity and reliability of healthy food knowledge and healthy food preferences scale for preschool children
Source: Front Pediatr. 2025 Apr 3;13:1507055. doi: 10.3389/fped.2025.1507055 (PMC12003418; doi:10.3389/fped.2025.1507055)

Healthy Food Knowledge

Point to the food that will help the doll become healthy, grow big, and strong.

- Kinche
- Cake

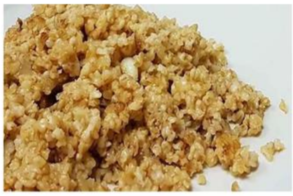

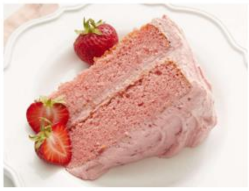

Point to the food that will help the doll become healthy, grow big, and strong.

- Banana
- Lollipop

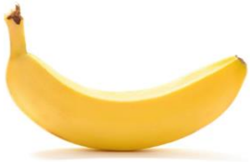

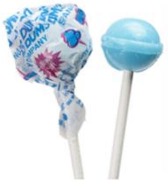

Point to the food that will help the doll become healthy, grow big, and strong.

- Doughnuts
- Macaroni

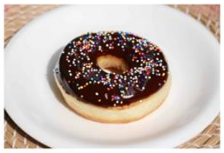

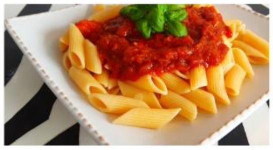

Point to the food that will help the doll become healthy, grow big, and strong.

- Rice
- Chips

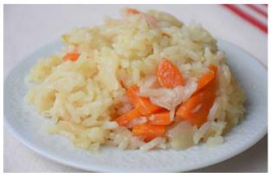

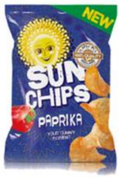

Point to the food that will help the doll become healthy, grow big, and strong.

- Enjera

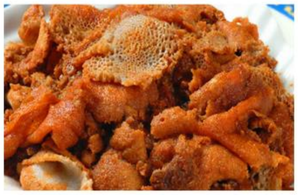

- Cookies

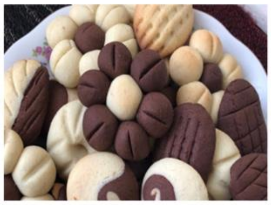

Point to the food that will help the doll become healthy, grow big, and strong.

- Milk

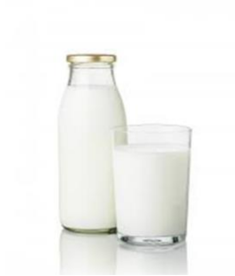

- Cola

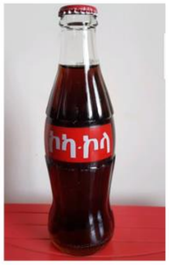

Point to the food that will help the doll become healthy, grow big, and strong.

- Water

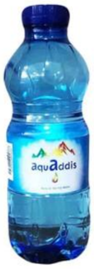

- Canned juice

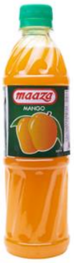

Point to the food that will help the doll become healthy, grow big, and strong.

- Mirinda

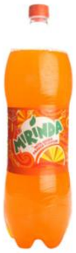

- Atmit

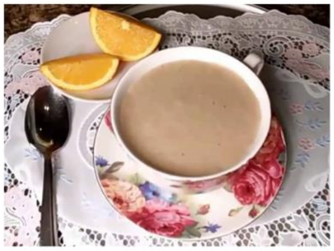

Point to the food that will help the doll become healthy, grow big, and strong.

- Fanta

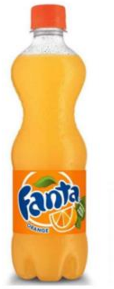

- Yogurt

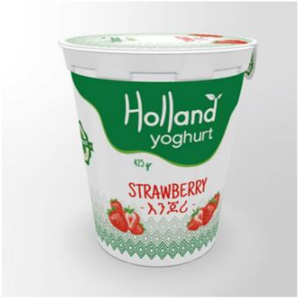

Healthy Food Preferences

Point to a food that you like the most

- Kinche

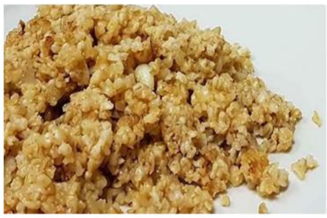

- Cake

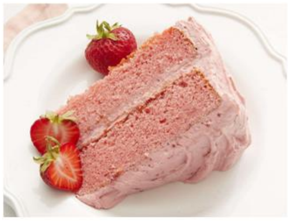

Point to a food that you like the most

- Banana

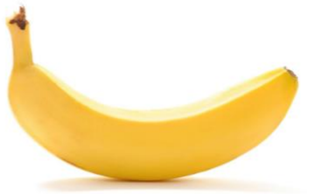

- Lollipop

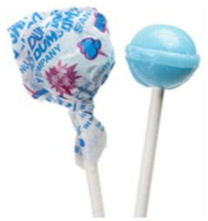

Point to a food that you like the most

• Doughnuts

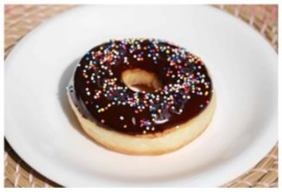

• Macaroni

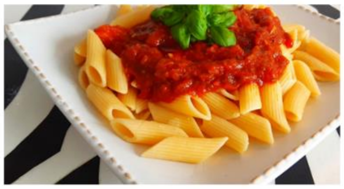

Point to a food that you like the most

• Rice

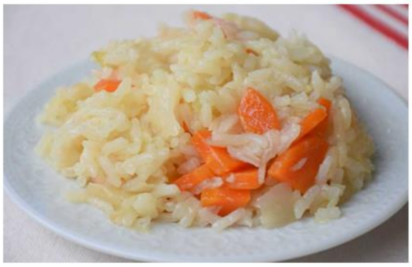

• Chips

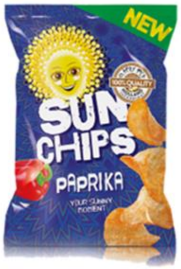

Point to a food that you like the most

• Enjera

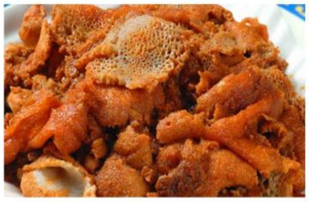

• Cookies

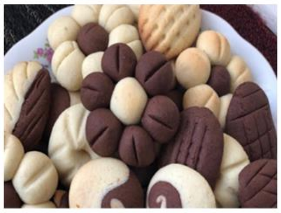

Point to a food that you like the most

• Milk

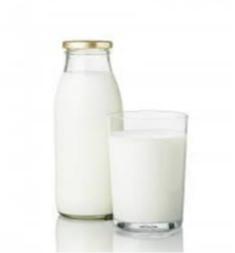

• Cola

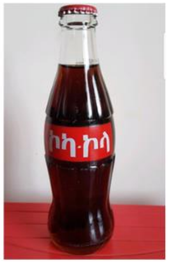

Point to a food that you like the most

• Water

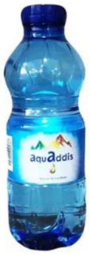

• Canned juice

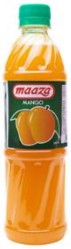

Point to a food that you like the most

• Mirinda

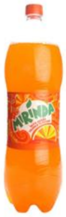

• Atmit

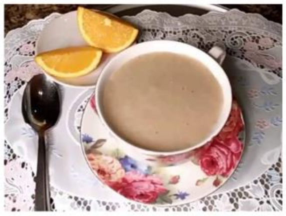

Point to a food that you like the most

• Fanta

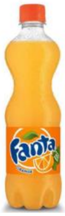

• Yogurt

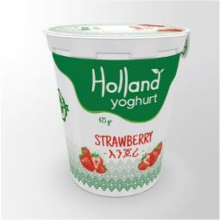

Supplement: Supplementary file 1 [file Datasheet1.pdf]
